# Kinematics Analysis of Male Runners via Forefoot and Rearfoot Strike Strategies: A Preliminary Study

**DOI:** 10.3390/ijerph192315924

**Published:** 2022-11-29

**Authors:** Chao-Fu Chen, Hui-Ju Wu, Chao Liu, Soun-Cheng Wang

**Affiliations:** 1 Physical Education College, Huaibei Normal University, Huaibei 235000, China; 2 Department of Athletic Sports, National Chung Cheng University, Minxiong 621301, Taiwan

**Keywords:** forefoot strike, rearfoot strike, exercise, kinematics, biomechanics

## Abstract

This study aimed to explore the kinematic characteristics of males using various foot landing strategies. The participants were fifteen male students from Physical Education College, Huaibei (non-professional runners, who did not have a fixed running landing strategy mode) (mean height = 178.20 cm; mean weight = 67.60 kg; mean age = 19.40 years). In this experiment, the running model of different foot landing strategies (forefoot strike, FFS and rearfoot strike, RFS) were analyzed using two high-speed cameras captured simultaneously at a sampling rate of 100 Hz. According to the results, the runners with better sports performance have shorter contact time, longer flight time, lower duty factor, larger stride angle, faster V COG, greater A COG, and knee and ankle angles which were crucial kinematics factors to enhance the running. Therefore, this study recommends that coaches or researchers can use photography to analyze novice runners who do not have a fixed landing pattern when running with RFS, the characteristics of running style was closely related to the flight times, and running with FFS was closely related to the stride angle.

## 1. Introduction

During running, there are commonly three modes of running: forefoot strike (FFS), midfoot strike (MFS), and rearfoot strike (RFS) mode [1,2,3,4]. Taking the vertical pressure center of the foot at the moment of the foot landing, if it falls between 0% and 33%, it is associated with the RFS; if it falls between 33% and 67%, it is pertinent to the MFS; and if it falls between 67% and 100%, it corresponds to the FFS [1,4,5,6]. Additionally, a survey of leisure runners reveals that about 69–95.1% of them employ RFS and MFS, and only 4.9–31% utilize the FFS, which demonstrates that most runners are accustomed to the RFS and MFS running modes, and only a few runners employ the FFS [7,8]; nevertheless, the evaluation approach through the pressure center of the ground foot is quite clear. As the actual evaluation is carried out, there are limitations in the actual implementation due to the unusualness of the force plate system.

In the past, researchers have pointed out that the main biomechanical factors influencing the running include anthropometry characteristics (weight and mass distribution, limb length, and Achilles tendon moment arm), and running style/gait pattern (stride, step rate, vertical oscillation, footstrike patterns, kinematics, kinetics, flexibility, and ground reaction force) [9,10]. However, biomechanical factors have been clearly classified into favorable and unfavorable categories. It is difficult for general running coaches and runners to truly apply the influencing factors of these running techniques due to the too many and complex variables to be considered. Past research had found that a runner’s duty factor was significantly correlated with running performance; duty factor was obviously an important indicator affecting running performance (duty factor (%) refers to the percentage of the time the foot contacts the ground and the time in the flight) [5,11,12]. In order to obtain the correct time and space variation data during running, coaches and researchers should frequently exploit high-speed cameras to record the changes in time and space variables such as step rate, stride, contact times, and flight times for action photography analysis during running [13].

Therefore, this study preliminarily analyzed the kinematic differences of male non-professional runners who did not have a fixed running landing strategy mode using different landing strategies, and gave runners who do not have a fixed landing mode to choose a suitable landing strategy. Using three-dimensional space photography to obtain kinematic-related parameters, the RFS, FFS, and kinematic-related data can be analyzed. Analysis and application integration of this kind of research will enrich sports theory and have specific potential application values as a technical improvement reference for coaches and athletes.

## 2. Experimental Design

### 2.1. Participants

Snowball sampling was used to recruit male students from the Physical Education College, Huaibei, and selected the fifteen voluntary participants (non-professional runners, and not have a fixed running landing strategy mode) (average height, weight, and age were 178.20 cm, 67.60 kg, and 19.40 years, respectively). The study complied with the Helsinki Declaration and was approved by Huaibei Normal University. Before the participants signed a letter of consent, the researcher will clearly explain the experimental process, research purpose and the action mode of the running and landing strategy, and let the participants fully understand.

### 2.2. Equipments and Data Collection

This experiment ran from November 2021 to June 2022, where each participant performed a total of 2 tests, the first with RFS and the second with FFS, on a treadmill at 10 km per hour and 160 steps per minute (Previous studies had pointed out that the average runner was about 145 to 160 steps per minute, and the professional runner was about 170 to 185 steps per minute, so this study selected 160 steps per minute.) [5,14]. The base and advanced variables of running and the kinematics of each movement during the running period were analyzed.

**Figure 1:**
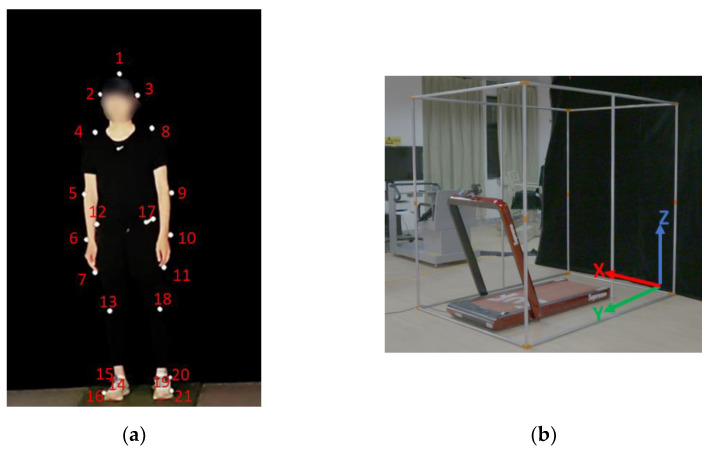
Experiment setup process. (**a**) Twenty one optical capture reflective markers. (**b**) Experimental frame.

After the participants warmed up for 10 min, the researchers adhered 21 optical capture reflective markers on the participant’s body (the head, right and left ear, middle fingertips, joints of shoulder, elbow, wrist, hip, knee, ankle, heels, and toe) (Figure 1a). A total of 21 landmarks and 14 segments were set, and the motion of the human body was assumed to be a rigid body structure with equal density of each limb segment. The limb segment parameters were built-in parameters of the Kwon3D motion analysis system (Table 1) [15,16,17]. Thereafter, the center of the treadmill was set as the origin, the size was set at 2.0 m × 1.5 m × 2.0 m (length × width × height) (Peak Motus), where the X of the global coordinate system represents the horizontal left and right of the space, Y represents the horizontal forward and backward of the space, and Z represents the vertical up and down direction (Figure 1b). This study used two Sony, PXW-FS7H high-speed cameras (sampling rate = 100 Hz, shutter speed = 1/1000 s, TOKYO, Japan), and an LED light was used as the camera synchronization signal, respectively at an angle of 45 degrees and extending 12 m to the side. The position of the center of the shooting coordinate frame, the optical axis of the lens, and its shooting range could cover the coordinate system. Finally, the action patterns of RFS and FFS running and landing strategies were analyzed.

### 2.3. Data Processing

**Figure 2:**
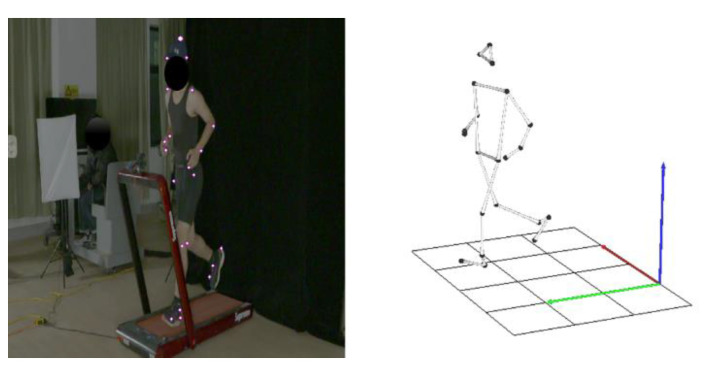
Motion analysis suite processes captured images.

The accuracy of kinematic data via high-speed cameras in this study was verified, the captured images were processed using the Kwon3D (Visol, Inc., Gwangmyeong-si, Kyonggi-do, Republic of Korea) motion analysis suite (Figure 2), and an optical auto-capture digitized the markers attached to the imaged joints. The original data was smoothed by a 4th order butterworth low pass filter to filter noise, the cutoff frequency was by a 6 Hz, and the three-dimensional spatial reconstruction error was 0.508157 cm. Finally, the required kinematic parameters were calculated.

Running base variable definitions: Step rate (spm) is the number of steps per unit time while running (steps per minute); stride (m) is the distance between the feet when running; contact times (s) is the time it takes for the foot to hit the ground, cushion and support the foot during running; flight times (s) is the time from when the foot (right foot) leaves the ground to when the other foot (left foot) touches the ground in the air during running [5,18,19].

Running advanced variable definition: Duty factor (%) refers to the percentage of the time the foot contacts the ground and the time in the flight (Equation (1)) [5,11,12]. Stride angle is the tangent angle when the running foot is off the ground. It is calculated based on the projection principle of the stride and the maximum moving height during the stride process. The maximum height of the stride is calculated based on the running flight time (Equation (2)) [5,20,21,22]. Through the force displacement linear formula (F = kX) of the spring, the vertical stiffness can be calculated by using the maximum ground reaction force (Fmax) and the vertical displacement of the body’s center of gravity (COG) (∆y) during running (Equation (3)), the leg stiffness can be calculated using Fmax and the vertical displacement of the leg length (ΔL) (Equation (4)) [5,23,24,25].

Duty factor (%) = t_c_/(t_c_ + t_f_) (1)

Maximum height = g(flight time)^2^/8 (2)

Vertical stiffness = Fmax/∆y(3)

Leg stiffness = Fmax/ΔL(4)

Note: t_c_ is contact times; t_f_ is flight times; g is the gravity (9.81 m s^−2^); Fmax = $\mathrm{mg}\frac{\pi }{2}\left(\frac{t_{f}}{t_{C}}+1\right)$; ∆L = $L-\sqrt{L^{2}-{\left(\frac{{\mathrm{vt}}_{c}}{2}\right)}^{2}+\Delta y}$; ∆y = $-\frac{F_{\max}t_{c}^{2}}{{m\pi }^{2}}+g\frac{t_{c}^{2}}{8}$; v is running speed; L is leg length.

Definition of various kinematic parameters of running: the COG velocity (V COG): the combined velocity, horizontal velocity and vertical V COG of the body; COG angle (A COG): the angle between the horizontal velocity and the combined velocity; the COG height (H COG): the vertical height relative to the ground; the angle of hip joint: the angle formed by three points of shoulder joint, hip joint and knee joint (when the human body is standing, the hip joint angle is 180 degrees); the angle of knee joint: the angle formed by the three points of the hip joint, knee joint and the ankle joint (when the human body is standing, the angle of the knee joint is 180 degrees); the angle of the ankle joint: the angle formed by the three points of the knee joint, the ankle joint and the toe (when the human body is standing, the ankle joint angle is 90 degrees).

**Figure 3:**
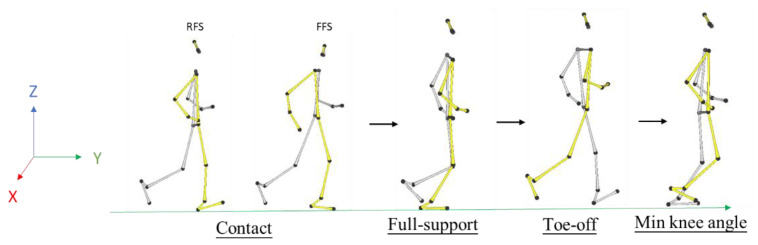
RFS and FFS running stages.

In this study, the RFS and FFS running phases was divided into four periods: (1) Contact phase. (2) Full-support phase. (3) Toe-off phase. (4) Min knee angle phase. (Figure 3) [2,3,4,5,6,26].

### 2.4. Statistical Analysis

Statistical analyses were performed using SPSS Version 17.0 for Windows. The Wilcoxon signed rank test was used for testing the base and advanced variables of RFS and FFS running and the kinematic differences of each movement during the running period (The data was not normally distributed). Multiple regression was used to analyze regression equations for RFS and FFS. The level of significance was set at α = 0.05. In addition, the effect size (ES) of RFS and FFS in each parameter was calculated by Cohen’s d as a practical evaluation of the quantitative results. The effect value is between 0.2–0.49 as a small effect, between 0.49–0.79 as a medium effect, and greater than 0.8 as a large effect [27]. Finally, the G*Power Version 3.1 software (Dusseldorf, Germany) was used to calculate the statistical power of RFS and FFS in each parameter, and the statistical significance level was set at Power = 0.8 [27].

## 3. Results

In the base variable, the flight time (s) FFS was significantly longer than RFS (z = −2.482, *p* = 0.013, ES = 0.632, Power =0.602). Among the advanced variables, the duty factor (%) FFS was significantly smaller than RFS (z = −2.594, *p* = 0.009, ES = 0.632, Power = 0.602). Among the advanced variables, FFS was significantly larger than RFS in stride angle (z = −2.552, *p* = 0.010, ES = 0.508, Power = 0.430) (Table 2).

In the kinematics of various movements during the running period, at the contact period, the V COG in FFS was significantly faster than RFS (z = −2.883, *p* = 0.004, ES = 1.046, Power = 0.955); the COG angle (A COG) in FFS was significantly larger than RFS (z = −2.924, *p* = 0.003, ES = 1.251, Power = 0.992); and the ankle joint angle in FFS was significantly larger than RFS (z = −4.003, *p* < 0.001, ES = 2.045, Power = 1.000). At the full-support period, the V COG in FFS was significantly faster than RFS (z = −3.007, *p* = 0.003, ES = 1.359, Power = 0.997); the H COG in FFS was significantly higher than RFS (z = −2.054, *p* = 0.040, ES = 0.800, Power = 0.800); the knee joint angle in FFS was significantly larger than RFS (z = −2.385, *p* = 0.017, ES = 0.913, Power = 0.892); and the ankle joint angle in FFS was significantly larger than RFS (z = −3.132, *p* = 0.002, ES = 1.402, Power = 0.998). At the toe-off period, the ankle joint angle in FFS was significantly larger than RFS (z = −2.987, *p* = 0.003, ES = 1.239, Power = 0.991). At the min knee angle period, the ankle joint angle in FFS was significantly larger than RFS (z = −3.256, *p* = 0.001, ES =1.391, Power = 0.998) (Table 3).

From the perspective of standardized regression coefficients for RFS, the β values of the three predictors entering the regression mode were −1.006, 0.162, and −0.020, respectively, indicating that the longer flight times, the shorter stride and the faster V COG in toe-off phase, can present a better duty factor (Table 4).

The standardized regression equation is as follows:

Duty factor (%) = −1.006 × Flight times + 0.162 × Stride − 0.020 × V COG in toe-off phase.(5)

From the perspective of standardized regression coefficients for FFS, the β values of the three predictors entering the regression mode were −0.784, 0.264, and −0.103, respectively, indicating that the larger the stride angle, the shorter the contact times and the larger A COG in the min knee angle phase, can present a better duty factor (Table 5).

The standardized regression equation as follows:

Duty factor (%) = −0.784 × Stride angle + 0.264 × Contact times − 0.103 × A COG in the min knee angle phase.(6)

## 4. Discussion

During running, the exploitation of the RFS or FFS strategies has always been a hot topic of discussion. This study specifically analyzes male college students in kinematics, using various foot-landing strategies, tested on a treadmill at the same speed and step rate. Additionally, in order to obtain correct spatiotemporal parameters of the running, coaches and researchers should employ high-speed cameras to analyze the variations of time and space variables such as step rate, stride, contact times, and flight times during running [5,6,11,28,29,30].

Ogueta-Alday [31] recorded the running spatiotemporal parameter variables for the half marathon results and found that in the peak speed (i.e., respiratory compensation and ventilatory threshold values) there are group differences in contact time, flight time, and stride. Runners with good running ability have shorter contact time, longer flight time, larger stride, and no significant difference in the step rate. In the cases of running at speeds 11, 13, and 15 km/h, runners with different abilities only have substantial differences in contact time. Nummela scholars [18] targeted 25 endurance athletes as participants. For running at eight different speeds, the contact time was shorter and the flight time was longer, and the maximum running speed of the runners during the 30-meter sprint was inversely proportional to the contact time of the feet (r = −0.52, *p* < 0.01), running at specific speeds with shorter contact times and longer flight times. However, when runners run at the same speed, running technology can be predicted based on the runner’s flight time [5,6,28,30]. In the current study, it is observed that the flight time(s) of the FFS is considerably longer than that of the RFS in the base variable (z = −2.482, *p* = 0.013, ES = 0.632, Power = 0.602). We can preliminarily estimate that runners employing the FFS strategy have longer flight times and better running technology.

Additionally, Morin et al. [32] discovered that a reduction in the step rate can substantially lead to the growth of flight times, an increase in step rate considerably lessens contact times, and the duty factor only increases significantly as the step rate reduces by −20% and −30%. A decrease or an increase in the step rate does not expressively alter the duty factor. Folland et al. [11] displayed that the running’s duty factor is a crucial technical condition affecting running performance such that the lower the duty factor, the better the running technology. It was observed in this advanced variable study that the duty factor (%) FFS would be significantly lower than the RFS (z = −2.594, *p* = 0.009, ES = 0.632, Power = 0.602). For runners utilizing the FFS strategy, it can be preliminarily predicted that the lower duty factor is commonly followed by the better running technology.

Additionally, having a larger stride angle is a pivotal kinematics factor for running technology [33]. Santos-Concejero et al. [22] conducted a study on 30 male runners with a 10 km running performance of 32.9 ± 2.7 min and maximum oxygen uptake of 63.1 ± 5.0 mL/kg/min. The performed study revealed that the ground runners using the FFS remarkably exhibited a larger stride angle than the ground runners with the RFS, and the running technology of the ground runners on the basis of the RFS was substantially better than that of the ground runners with the FFS. Due to the remarkable difference in the 10 km running performance of the two groups of participants in the current study, RFS-based runners were 34.3 ± 3.2 min, and FFS-based runners were 31.7 ± 1.4 min, resulting in a considerable discrepancy in stride angle. The reasons still should be further clarified; nevertheless, in this advanced variable scrutiny, it is observed that at the same speed and step rate, the stride angle FFS is remarkably larger than that of the RFS (z = −2.552, *p* = 0.010, ES = 0.508, Power = 0.430). We can preliminarily estimate that for runners based on the FFS strategy, the larger the stride angle, the better the running technology. Additionally, having greater leg stiffness is a vital factor in enhancing the running technology [33]. In general, direct measuring vertical (kvert) and leg stiffness (kleg) values during running is a simple approach to exploring leg stiffness [25]. However, no substantial discrepancy in the vertical stiffness and the leg stiffness was observed between the RFS and the FFS in this advanced variable study, and this study supposes that runners may have about the same ability but found that the exercise performance of the ground runners based on the FFS would be more effective.

In the kinematics of each movement during the running period, it was also detected that the FFS height of the ankle joint angle would be significantly larger than that of the RFS during the contact period, full-support period, toe-off period, and min knee angle period. Santos-Concejero et al. [34] explained that the phenomenon of increasing the stride angle during running led to a specific manifestation of the flick or effect, in order to enhance the efficiency of energy transfer under the conditions of minimum contact time. However, the current investigation also observed that the FFS H COG during the contact and full-support periods would be considerably higher than that of the RFS. Further, the A COG FFS height during the contact period was remarkably larger than that of the RFS, and the H COG FFS during the full-support period was significantly higher than that of the RFS. The knee joint angle of the FFS was substantially larger than that of the RFS at the stage. This is chiefly attributed to the growth of the V COG and the knee extension pattern during foot immobilization and propulsion during touchdown and standing [1,28,30,35]. In particular, the function of increasing the stride angle can provide the benefits of the stretch-shortening cycle of the leg muscles and enhance the energy transfer in running [1,29,35]. For excellent runners, having a larger stride angle actually provides a crucial condition for enhancing running technology.

From the multiple regression analysis of RFS, it can be found that the amount of explanation for a variable of “flight time” was as high as 97.3%, and the β value was negative, indicating that the longer the flight time, the better the duty factor of the running can be presented, which in turn improves the performance of using the RFS. Also mentioned in the past literature, discovered that a reduction in the step rate can substantially lead to the growth of flight times, an increase in step rate considerably lessens contact times, and the duty factor only increases significantly as the step rate reduces by −20% and −30%. A decrease or an increase in the step rate does not expressively alter the duty factor [32]. Therefore, it was recommended that runners who were accustomed to using the RFS can increase their flight time through training and enhance their running performance.

From the multiple regression analysis of FFS, it can be found that the explanation amount of a variable of “stride angle“ was as high as 94.0%, and the β value was negative, indicating that the larger the stride angle, the better the duty factor of running can be presented, and then the improvement performance using the FFS. The past literature has mentioned that having a larger stride angle was a key kinematic factor for running technique [33]. Therefore, it was recommended that runners who were accustomed to using the FFS can increase the stride angle through training to enhance their running performance. 

This study was a preliminary study, and the participants were male non-professional runners, so the results of the study were only suitable for male non-professional runners, and the relationship between injury and physical performance was not discussed in depth. Furthermore, the participants did not have a fixed running landing strategy mode, the MFS was a running mode that was difficult to control, so this study only explores the difference between FFS and RFS. The above were all limitations of this study. It was suggested that future research can further explore the analysis of different foot landing strategies of female or professional runners.

## 5. Conclusions

This was a preliminary study that allows coaches or researchers to understand the kinematic analysis of runners through camera footage. The study found that runners with better sports performance have shorter contact time, longer flight time, lower duty factor, larger stride angle, faster V COG, greater A COG, and knee and ankle angles which were crucial kinematics factors to enhance the running. In addition, based on the results of the study, it was suggested that coaches or researchers can use photography to analyze novice runners who do not have a fixed landing pattern, when running with RFS, the characteristics of running style was closely related to the flight times, and running with FFS was closely related to the stride angle. 

## Figures and Tables

**Table 1:** The limb segment parameters.

	Center of Gravity of Segment (Proximal)	Mass of Segment	Moment of Inertia-Frontal Axis	Moment of Inertia-Sagittal Axis	Moment of Inertia-Vertical Axis
	(CM) (%)	(Mass) (%)	(Ixx) (kg·cm^2^)	(Iyy) (kg·cm^2^)	(Izz) (kg·cm^2^)
Trunk	47.66	46.84	25.55	31.82	15.17
Head	50	8.26	25.82	26.47	28.36
Upper arm	43.6	3.25	29.98	30.69	12.08
Antebrachium	43	1.87	28.8	29.23	10.35
Hand	46.8	0.65	46.28	50.84	25.59
Thigh	43.3	10.5	32.04	31.51	13.58
Shank	43.4	4.75	29.18	29.18	7.67
Foot	50	1.43	25.69	26.94	12.45

Note: Ixx, Iyy, and Izz were the moments of inertia about the X, Y, and Z axes, respectively.

**Table 2:** Male running base and advanced variable parameters. (Mean ± SD) (*N* = 15).

	RFS			FFS			*z*		*d*	Effect Size	Power
Base variable											
Stride (m)	1.02	±	0.06	1.03	±	0.07	−0.715		0.153	-	0.080
Contact times (s)	0.32	±	0.03	0.31	±	0.03	−1.610		0.333	small	0.216
Flight times (s)	0.05	±	0.02	0.06	±	0.01	−2.482	*	0.632	medium	0.602
Advanced variable											
Duty factor (%)	0.87	±	0.06	0.84	±	0.03	−2.594	**	0.632	medium	0.602
Stride angle (deg)	0.73	±	0.71	1.01	±	0.32	−2.552	*	0.508	medium	0.430
Vertical stiffness(kN/m)	21.15	±	3.43	20.50	±	4.09	−0.726		0.172	-	0.093
Leg stiffness (kN/m)	7.51	±	1.99	7.83	±	1.74	−0.892		0.171	-	0.092

* *p* < 0.05; ** *p* < 0.01; RFS: rearfoot strike; FFS: forefoot strike; *z* = $\left(\bar{x}-\mu \right)∕\sigma_{\bar{x}}$; Cohen’s d was an effect size = (M2-M2)/pooled standard deviation.

**Table 3:** Kinematics of various movements in male running period. (Mean ± SD) (*N* = 15).

	RFS			FFS			*z*		*d*	Effect Size	Power
Contact											
V COG (m/s)	0.57	±	0.09	0.65	±	0.06	−2.883	**	1.046	large	0.955
A COG (deg)	−82.23	±	5.24	−88.48	±	4.74	−2.924	**	1.251	large	0.992
H COG (m)	102.77	±	3.49	100.16	±	3.72	−1.950		0.724	medium	0.719
Hip joint (deg)	160.31	±	13.37	167.01	±	4.77	−1.431		0.667	medium	0.648
Knee joint (deg)	160.16	±	9.27	160.73	±	5.46	−0.353		0.075	-	0.058
Ankle joint (deg)	89.24	±	7.03	106.60	±	9.73	−4.003	**	2.045	large	1.000
Full-support											
V COG (m/s)	0.44	±	0.17	0.64	±	0.12	−3.007	**	1.359	large	0.997
A COG (deg)	57.19	±	77.59	94.68	±	4.24	−1.390		0.682	medium	0.667
H COG (m)	95.26	±	4.51	98.56	±	3.70	−2.054	*	0.800	large	0.800
Hip joint (deg)	166.88	±	11.22	169.12	±	4.75	−0.270		0.260	small	0.150
Knee joint (deg)	141.16	±	7.57	149.03	±	9.56	−2.385	*	0.913	large	0.892
Ankle joint (deg)	74.63	±	6.11	87.51	±	11.47	−3.132	**	1.402	large	0.998
Toe-off											
V COG (m/s)	0.25	±	0.12	0.22	±	0.10	−0.560		0.272	small	0.160
A COG (deg)	−41.73	±	59.41	−58.86	±	32.10	−0.767		0.233	small	0.130
H COG (m)	104.02	±	3.66	102.44	±	4.21	−1.099		0.401	small	0.291
Hip joint (deg)	165.06	±	7.37	162.74	±	6.47	−1.265		0.335	small	0.218
Knee joint (deg)	145.43	±	6.44	150.02	±	8.51	−1.472		0.608	medium	0.569
Ankle joint (deg)	103.44	±	6.72	115.96	±	12.61	−2.987	**	1.239	large	0.991
Min knee angle											
V COG (m/s)	0.26	±	0.14	0.30	±	0.14	−0.850		0.286	small	0.172
A COG (deg)	81.78	±	70.50	87.15	±	63.28	−0.518		0.080	-	0.059
H COG (m)	96.90	±	3.59	94.96	±	4.15	−1.472		0.500	medium	0.419
Hip joint (deg)	161.72	±	15.93	166.33	±	6.83	−0.684		0.376	small	0.262
Knee joint (deg)	95.95	±	16.26	99.95	±	15.35	−0.270		0.253	small	0.144
Ankle joint (deg)	87.19	±	5.25	100.90	±	12.91	−3.256	**	1.391	large	0.998

* *p* < 0.05; ** *p* < 0.01; RFS: rearfoot strike; FFS: forefoot strike; *z* = $\left(\bar{x}-\mu \right)∕\sigma_{\bar{x}}$; Cohen’s d was an effect size = (M2-M2)/pooled standard deviation.

**Table 4:** Multiple linear regressions for RFS.

Model	R	R Square	ΔF	B	Beta (*β*)
(constant)				0.856	
Flight times	0.986	0.973	469.470 **	−2.889	−1.006
Stride	1.000	0.999	457.968 **	0.151	0.162
V COG in the toe-off phase	1.000	1.000	13.511 **	−0.010	−0.020

** *p* < 0.01.

**Table 5:** Multiple linear regressions for FFS.

Model	R	R Square	ΔF	B	Beta (*β*)
(constant)				0.829	
Stride angle	0.970	0.940	205.107 **	−0.068	−0.784
Contact times	0.995	0.990	57.383 **	0.262	0.264
A COG in the min knee angle phase	0.999	0.997	27.064 **	0.000	−0.103

** *p* < 0.01.

## Data Availability

Not applicable.
